# Detection of circulating tumor cells by p75NTR expression in patients with esophageal cancer

**DOI:** 10.1186/s12957-016-0793-9

**Published:** 2016-02-20

**Authors:** Tetsuji Yamaguchi, Tomoyuki Okumura, Katsuhisa Hirano, Toru Watanabe, Takuya Nagata, Yutaka Shimada, Kazuhiro Tsukada

**Affiliations:** Department of Surgery and Science, Graduate School of Medicine and Pharmaceutical Sciences, University of Toyama, 2630 Sugitani, Toyama City, Toyama 930-0194 Japan; Department of Nanobio Drug Discovery, Graduate School of Pharmaceutical Sciences, Kyoto University, 46-29 Yoshida-Shimo-Adachi-cho, Sakyo-ku, Kyoto 606-8501 Japan

**Keywords:** Esophageal cancer, Circulating tumor cells, Cancer stem cells, Flow cytometry, p75NTR

## Abstract

**Background:**

The p75 neurotrophin receptor (p75NTR) is a cancer stem cell (CSC) marker in esophageal squamous cell carcinoma (ESCC). This study aimed to assess the use of p75NTR in detecting circulating tumor cells (CTCs) in ESCC.

**Methods:**

Peripheral blood mononuclear cell expression of epithelial cell adhesion molecule (EpCAM) and p75NTR was detected in 23 ESCC patients (13 received chemo- or chemoradiotherapy and 10 received curative surgery) and 10 healthy controls by flow cytometry.

**Results:**

EpCAM + p75NTR+ cell counts (average ± SD) were significantly higher in patients (*n* = 23, 16.0 ± 18.3) compared to controls (*n* = 10, 0.4 ± 0.9, *p* = 0.013). The sensitivity and specificity to differentiate ESCC patients from controls were 78.3 and 100 % (cut-off value 4.0), respectively. EpCAM + p75NTR+, but not EpCAM + p75NTR− cell counts, correlated with clinically diagnosed distant metastasis (*n* = 13, *p* = 0.006) and pathological venous invasion in resected primary tumors (*n* = 10, *p* = 0.016). Malignant cytology was microscopically confirmed in isolated EpCAM + p75NTR+ cells with immunocytochemical double staining.

**Conclusions:**

p75NTR is suggested to be a useful marker for clinically significant CTCs, which exhibit highly metastatic features in ESCC.

## Background

Esophageal squamous cell carcinoma (ESCC) is a leading cause of cancer-related deaths worldwide [1]. Despite advances in its diagnosis and multimodal therapies, the prognosis for ESCC patients remains poor with post-operative 5-year survival rates of 42 %, due to high incidences of metastasis [2, 3].

Lately, attention has focused on the proportion of circulating tumor cells (CTCs) as an early marker of metastasis [4]. The CTC detection method most extensively used is based on immunomagnetic enrichment with epithelial cell adhesion molecule (EpCAM) and cytokeratin antibodies for subsequent immunological identification [5, 6]. Quantification of CTCs using this method has established CTCs as an independent prognostic factor in patients with advanced colorectal [7], breast [8], and prostate cancer [9].

Other studies have identified cancer stem cells (CSCs), which are defined as a small fraction of tumor cells capable of self-renewal, tumorigenicity, and drug resistance [10, 11]. CSCs are also responsible for metastasis through processes such as epithelial mesenchymal transition, invasion into vessels, circulation, and tumor initiation [12, 13].

Moreover, reports suggest that a percentage of CTCs have characteristics reminiscent of CSCs, which are termed circulating tumor stem cells (CTSCs) [14]. Compared to CTCs, CTSCs are potentially a more accurate prognostic factor since cancer growth is dependent on CSCs [15].

In ESCC, several cell surface antigens have been reported to be CSC markers, such as p75 neurotrophin receptor (p75NTR) [16–18] and CD44 [19]. A recent report demonstrated that p75NTR-positive cells in ESCC cell lines showed significantly higher colony formation, enhanced tumor formation in mice, and greater chemoresistance, along with stronger expression of epithelial mesenchymal transition (EMT)-related genes [18]. However, detection of CTCs using CSC markers in ESCC has not previously been reported. The aim of this study was to detect CTCs in the peripheral blood of ESCC patients using combined expression of EpCAM, CD44, and p75NTR to assess its clinical significance.

## Methods

### Patients

This study included a series of 23 primary ESCC patients who consulted our hospital between January and December 2014. Nineteen of 23 patients were men, and the median age was 62.3 (range 38–88) years. Ten patients underwent R0 resection, while 13 patients received chemotherapy (CT) or chemoradiation therapy (CRT) without resection. Clinicopathological classifications were determined according to the criteria of the Union for International Cancer Control (UICC)-TNM Classification of Malignant Tumors Staging System (seventh edition). In the patients who underwent CT or CRT, 1, 4, 2, 1, and 5 patients were clinically diagnosed as cStageIB, cStageIIIA, cStageIIIB, cStageIIIC, and cStageIV (intramural metastasis to the stomach 3; bone 1; distant lymph node 1), respectively. In the patients who underwent surgery, 2, 5, 2, and 1 patients were pathologically diagnosed as stage IA, stage IIA, stage IIIA, and stage IV (T3N2M1: intramural metastasis to the stomach), respectively. Three patients presented with lymph node metastasis and lymph vessel invasion, and three presented with venous invasion.

Ten healthy volunteers were also recruited as negative controls. Subjects provided informed written consent prior to commencing the study, and all were enrolled following Institutional Review Board approval (no. 20-75).

### Immunohistochemistry

Formalin fixed-paraffin embedded sections were prepared for examination. Antibodies required for immunohistochemical analysis included anti-cytokeratin OSCAR IVD (dilution 1:200) and anti-human p75NTR monoclonal antibody (NGFR5; dilution 1:100) (Abcam Ltd., Cambridge, UK). Immunostaining was performed using Envision Plus kits/HRP/DAB (Dako Cytomation, Kyoto, Japan), as recommended by the supplier. Counterstaining was performed using Mayer’s hematoxylin (Dako Cytomation, Kyoto, Japan).

### Peripheral blood sample processing and analysis

Blood samples were collected from ten patients who underwent R0 resections on the day before surgery. As for the 13 patients who received CT or CRT, blood samples were obtained before the start of the therapy. Blood samples were collected from each donor in ethylene-diamine-tetra-acetic acid (EDTA)-2K Vacutainer Tubes (TERUMO, Tokyo, Japan). Specimens were maintained at 4 °C and processed within 4 h of phlebotomy. Mononuclear cells were enriched from 3 mL of peripheral blood using LymphoPrep™ (Nycomed AS, Oslo, Norway). These were incubated in the presence of allophycocyanin (APC)-conjugated monoclonal mouse anti-human EpCAM [clone HEA (human epithelial antigen)-125, dilution 1:100], fluorescein isothiocyanate (FITC)-conjugated monoclonal mouse anti-human p75 neurotrophic receptor (p75NTR, clone ME20.4-1.H4, dilution 1:50), FITC-conjugated monoclonal mouse anti-human CD44 (clone BD105, dilution 1:100, Miltenyi Biotec), or isotype-matched APC/FITC-conjugated antibodies (clone IS5-21F5, dilution 1:50) (Miltenyi Biotec, Bergisch Gladbach, Germany) at 4 °C for 30 min. Samples were analyzed on the fluorescence-activated cell sorting (FACS)Canto II flow cytometer and sorted on a FACS Aria II flow cytometer using BD FACS Diva software (BD Biosciences, San Jose, CA, USA). Cells were stained with 7-aminoactinomycin D (7-AAD; Bio-Rad Laboratories, CA, USA) to exclude any dead cells.

### Flow cytometric detection of cultured cells mixed with blood samples

Human ESCC cell lines (KYSE30 and KYSE140) were established by Shimada and colleagues and cultured in Ham’s F12/RPMI-1640 (Wako, Osaka, Japan) with 2 % fetal calf serum (Gibco; Grad Island, NY, USA) according to a previously published method [20]. For the accuracy assay, 0, 10, 100, and 500 of KYSE cells were mixed with 3 mL each of peripheral blood samples obtained from the healthy volunteers.

### Cytological examination of the sorted cells

Sorted EpCAM + p75NTR+ cells were centrifuged at 250× *g* for 5 min using a Cytospin™ chamber (Stat Spin, Beckman Coulter, Tokyo, Japan) and evaluated using a transmitted light microscope (BX61/DP70, Olympus, Tokyo, Japan).

### Culture of the sorted cells

Sorted EpCAM + p75NTR+ cells were resuspended with 100 μl of Ham’s F12/RPMI-1640 with 2 % fetal calf serum and seeded onto one of the 96-well cell culture plates and cultured at 37 °C in a humidified atmosphere of 5 % carbon dioxide in air for 14 days.

### Statistical analysis

Statistical analyses were performed using JMP v.11 (SAS Institute Inc., Cary, NC, USA). Chi-square and Fisher’s exact tests were performed, and probability (*p*) values of less than 0.05 were deemed statistically significant. Receiver operating characteristic (ROC) curves and the area under the curve (AUC) were used to assess the feasibility of using EpCAM+ cells and EpCAM+/p75NTR+ cell counts as a measure of CTC numbers in patients with ESCC.

## Results

### Accuracy assay for detecting CTCs

**Fig. 1:**
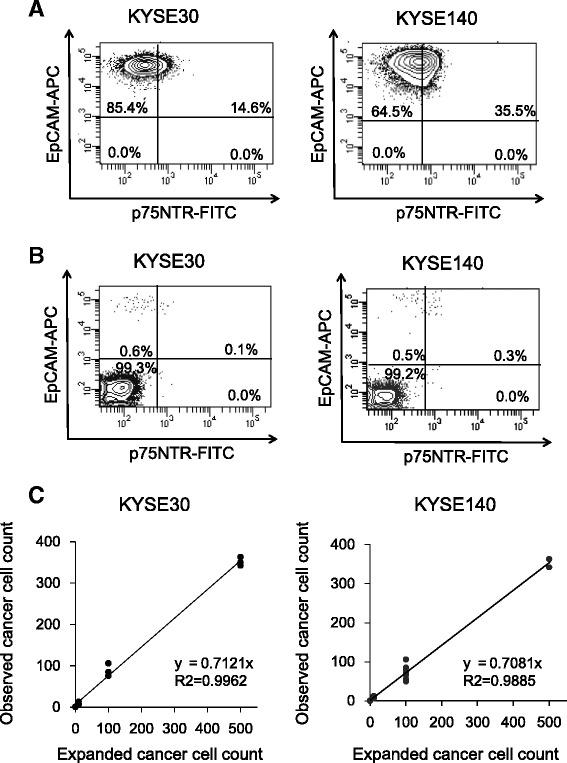
**a** KYSE30 and KYSE140 cells co-stained with anti-EpCAM-APC and anti-p75NTR-FITC were analyzed by two-color flow cytometry. Data of percentages of cells in each quadrant are shown. **b** Mononuclear cells from 3 mL peripheral blood from a healthy control, in which 100 of KYSE30 and KYSE140 cells were mixed and co-stained with anti-EpCAM-APC and anti-p75NTR-FITC, were analyzed by two-color flow cytometry. **c** Representative result of the number of spiked KYSE30 and KYSE140 cells plotted against the observed number of cells using flow cytometry is shown

EpCAM expression was detected in 100 and 100 % of KYSE30 and KYSE140, respectively. Within the EpCAM+ cells, 14.6 and 35.5 % of KYSE30 and KYSE140 cells, respectively, were p75NTR+ (Fig. 1a). EpCAM+ cells were detected in the peripheral blood of healthy volunteers, in which 0, 10, 100, and 500 of KYSE30 and KYSE140 cells were mixed beforehand (Fig. 1b). Regression analysis on the numbers of observed EpCAM+ versus the expected KYSE30 and KYSE140 cells produced a slope of 0.71 (95 % confidence interval (CI) 0.68–0.74) and 0.70 (95 % CI 0.67–0.74), respectively. The intercepts were 4.8 (95 % CI −4.4–14.0) and 1.4 (95 % CI −4.6–7.4) with the correlation coefficients (*R*^2^) of 0.997 and 0.988, respectively (Fig. 1c).

### Detection of CTCs in ESCC patients based on EpCAM expression

**Fig. 2:**
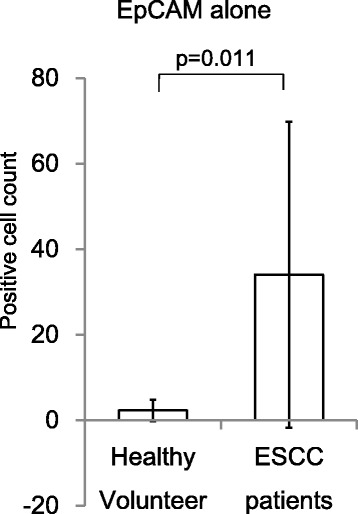
Mean numbers of EpCAM+ cells from 3 mL peripheral blood of ESCC patients and controls. *Error bars* represent the standard error of the mean

EpCAM+ cells were detected in 5 of 10 (50.0 %) healthy controls and 22 of 23 (95.7 %) ESCC patients with cell counts (average ± SD) of 2.3 ± 2.5 and 34.0 ± 35.8, respectively (*p* = 0.011, Fig. 2). Based on the ROC curve analysis to differentiate ESCC patients from controls, the largest AUC value for EpCAM+ cell counts was 7.0 with optimal sensitivity and specificity measurements of 86.9 and 100 %, respectively.

### Detection of CTCs in ESCC patients based on p75NTR expression

**Fig. 3:**
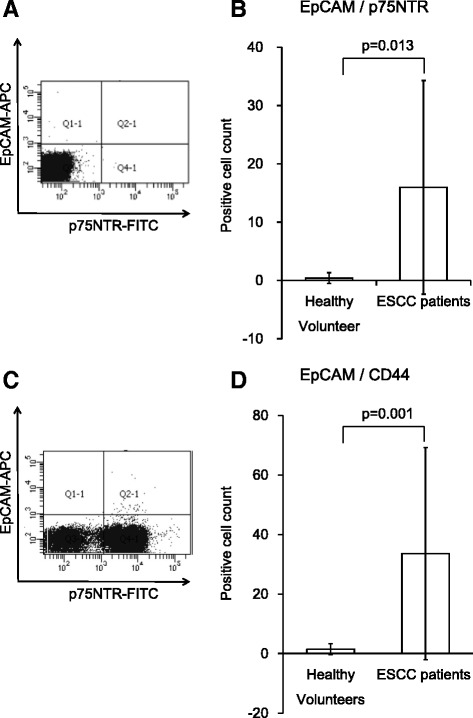
**a** Mononuclear cells from 3 mL peripheral blood were co-stained with anti-EpCAM-APC and anti-p75NTR-FITC and analyzed by two-color flow cytometry. *Quadrant markers* were set according to isotype-matched controls. **b** Mean numbers of p75NTR+ CTCs from 3 mL peripheral blood of ESCC patients and controls. *Error bars* represent the standard error of the mean. **c** Mononuclear cells from 3 mL peripheral blood were co-stained with anti-EpCAM-APC and anti-CD44-FITC and analyzed by two-color flow cytometry. *Quadrant markers* were set according to isotype-matched controls. **d** Mean numbers of CD44+ CTCs from 3 mL peripheral blood of ESCC patients and controls. *Error bars* represent the standard error of the mean

EpCAM + p75NTR+ cells were detected using two-color flow cytometry (Fig. 3a) in 2 of the 10 (20 %) controls and 20 of 23 (86.9 %) ESCC patients, with cell counts (average ± SD) of 0.4 ± 0.9 and 16.0 ± 18.3, respectively (*p* = 0.013, Fig. 3b).

The proportion of EpCAM + p75NTR+ cells in EpCAM+ cells (average ± SD) was 56.7 ± 39.6 % (range 5.6–100.0 %). Based on the ROC curve analysis to differentiate ESCC patients from controls, the largest AUC value for EpCAM + p75NTR+ cell counts was 4.0 with optimal sensitivity and specificity measurements of 78.3 and 100 %, respectively. The relationship between CTC counts and various clinicopathological features in the 13 patients who underwent CT or CRT is presented in Table 1. The mean EpCAM + p75NTR+ cell counts correlated with clinically diagnosed distant metastasis (*p* = 0.003), while EpCAM + p75NTR− cell counts did not show relationship with any of these pathological factors. The relationships between CTC counts and various clinicopathological factors in the ten patients who underwent R0 resection are presented in Table 2. The mean EpCAM + p75NTR+ cell counts correlated with pathological venous invasion (*p* = 0.016), while EpCAM + p75NTR− cell counts did not show relationship with any of the pathological factors.

**Table 1:** Relationship between the clinical features and mean EpCAM + p75NTR+ or EpCAM + p75NTR− CTC counts in patients who received chemotherapy or chemoradiotherapy

Characteristics	Number	EpCAM + p75NTR+ (average ± SD)	*p* value	EpCAM + p75NTR− (average ± SD)	*p* value
Male/female	9/4	16.4 ± 19.7/14.0 ± 14.4	0.764	26.0 ± 34.9/13.3 ± 14.1	0.504
Age ≥65/>65	5/8	22.8 ± 11.6/11.3 ± 11.9	0.114	38.2 ± 44.0/12.06 ± 11.7	0.131
Ce-Ut/Mt-Ae	2/11	19.0 ± 20.5/15.0 ± 12.2	0.666	2.0 ± 2.8/25.7 ± 31.4	0.325
T 0–2/3–4	1/12	7.0/16.4 ± 13.0	0.501	0/23.9 ± 30.6	0.468
N 0/1–3	1/12	7.0/16.4 ± 13.0	0.501	0/23.9 ± 30.6	0.468
M 0/1	8/5	7.1 ± 5.9/29.4 ± 6.4	*0.003	19.5 ± 20.8/26.2 ± 43.7	0.713
Stages 1–2/3–4	1/12	7.0/16.4 ± 13.0	0.501	0/23.9 ± 30.6	0.468

*CTC* circulating tumor cell, *SD* standard deviation

*p < 0.05

**Table 2:** Relationship between the clinicopathological features and mean EpCAM + p75NTR+ or EpCAM + p75NTR− CTC counts in patients who underwent surgery

Characteristics	Number	EpCAM + p75NTR+ (average ± SD)	*p* value	EpCAM + p75NTR− (average ± SD)	*p* value
Male/female	10/0	16.5 ± 25.2/–	–	12.0 ± 31.9/–	–
Age ≥65/>65	5/5	19.6 ± 11.9/13.4 ± 11.9	0.722	20.4 ± 45.6/23.6 ± 5.4	0.437
Ce-Ut/Mt-Ae	1/9	8.0/17.4 ± 26.6	0.745	0/13.3 ± 33.5	0.716
pT 0–2/3–4	4/6	2.0 ± 2.8/26.2 ± 29.3	0.146	27.0 ± 50.1/2.0 ± 4.9	0.246
pN 0/1–3	7/3	14.9 ± 27.7/20.3 ± 23.1	0.773	0.8 ± 2.3/38.0 ± 55.7	0.090
ly 0/1–2	7/3	14.9 ± 27.7/20.3 ± 23.1	0.773	0.8 ± 2.3/38.0 ± 55.7	0.090
v 0/1–2	7/3	5.0 ± 4.2/43.3 ± 35.6	*0.016	0.8 ± 2.3/38.0 ± 55.7	0.090
pStages 1–2/3–4	7/3	15.7 ± 27.2/18.3 ± 25.1	0.891	15.4 ± 38.2/4.0 ± 6.9	0.632

*CTC* circulating tumor cell, *ly* lymphatic invasion, *v* venous invasion, *SD* standard deviation

*p < 0.05

### Detection of CTCs in ESCC patients based on CD44 expression

EpCAM + CD44+ cells were detected in 5 of 10 (50.0 %) healthy controls and 22 of 23 (95.7 %) ESCC patients with cell counts (average ± SD) of 1.5 ± 1.7 and 33.6 ± 35.6, respectively (*p* = 0.001, Fig. 3c, d). The proportion of EpCAM + CD44+ cells in EpCAM+ cells (average ± SD) was 98.6 ± 3.1 % (range 90.5–100 %), indicating that almost all the EpCAM+ cells were CD44+ and thus it was not useful to differentiate subpopulations in EpCAM+ CTCs.

### Cytology of sorted EpCAM + p75NTR+ cells

**Fig. 4:**
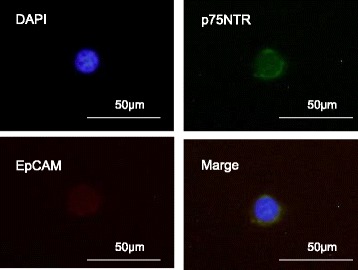
Representative EpCAM+ and p75NTR+ cell sorted from 3 mL peripheral blood in a patient with esophageal cancer using the fluorescence-activated cell sorting (FACS) Aria II flow cytometer. The images show an overlay of DAPI (*blue*), p75NTR (*green*), and EpCAM (*red*)

We evaluated sorted EpCAM + p75NTR+ cells from the peripheral blood of an ESCC patient microscopically. A representative figure of cells staining positive for EpCAM-APC (red) and p75NTR-FITC (green), in the cell membranes, is provided in Fig. 4. The cell represented is a mononuclear cell, 35 μm in diameter with a high nucleocytoplasmic ratio, which led cytopathologists to establish a diagnosis of malignant cytology.

We could not establish primary culture of the EpCAM + p75NTR+ cells sorted from any of the 23 examined patients (data not shown).

### Expression of p75NTR in the primary tumor of resected ESCC specimens

**Fig. 5:**
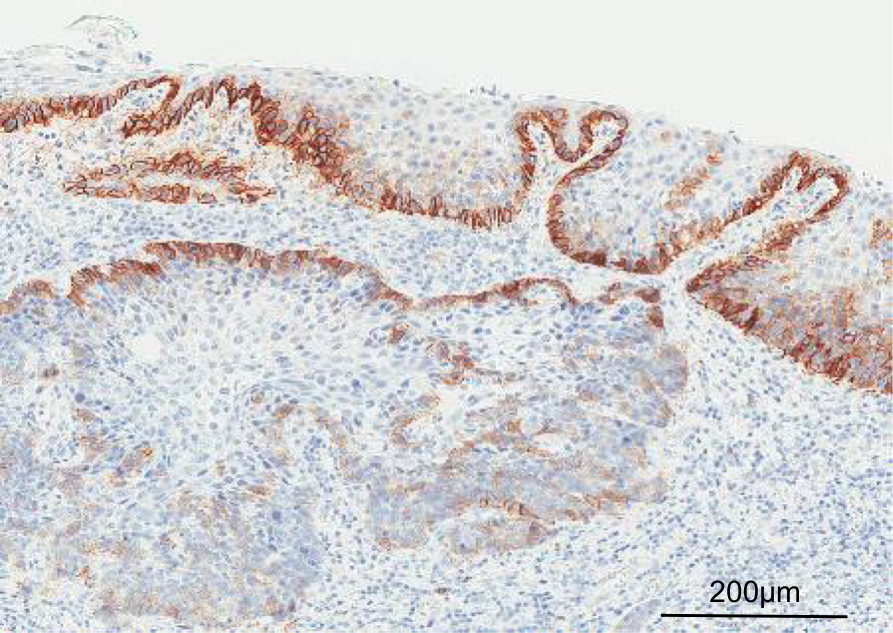
Representative findings of immunohistochemical analysis of p75NTR expression in resected ESCC tissue specimens

The expression of p75NTR was detected in five (50.0 %) tumors, in which the first few layers nearest to the tumor’s infiltrative margin exhibited positive staining (Fig. 5). There was no relationship between p75NTR expressions in primary tumors versus EpCAM+ cells of the peripheral blood.

## Discussion

In this study, we detected CTCs by flow cytometry based on the combined expression of EpCAM and p75NTR in patients with ESCC. Our results demonstrated that the EpCAM+ cell count in the peripheral blood of patients was significantly higher than that of controls, reflecting a tumor-bearing state, and thus indicating successful detection of CTCs using this method. Our results were consistent with those in previously published reports of patients with metastatic lung cancer (CK + CD45− CTCs detected by flow cytometry in all examined patients, with significantly higher cell counts than controls) [21, 22]. We revealed that detecting a combination of EpCAM and p75NTR expression was more accurate as a diagnostic marker than EpCAM detection alone.

In addition, our results highlighted that EpCAM + p75NTR+, but not EpCAM + p75NTR− CTC counts, correlated with clinically diagnosed distant metastasis and pathological venous factors in the resected primary tumor, demonstrating the highly invasive and metastatic potential of CTCs with p75NTR expression.

Recent reports have identified CTSCs, which have CSC-like potential and enhanced metastatic capacity in various solid tumors, such as breast [23], colon [24], and hepatocellular carcinoma [25], using a combination of EpCAM and CSC markers in the each tumor (e.g., CD44 or CD133). Further reports have also demonstrated CTSCs to be useful markers for the diagnosis, treatment responsiveness, and prognosis of patients with breast [26] and gastric cancer [27]. To the best of our knowledge, this is the first report to detect CTCs expressing a CSC marker in ESCC patients using flow cytometry.

In this study, we also detected CTCs with combined expression of EpCAM and CD44, which is one of the other proposed CSC markers in ESCC [19]; however, almost all the EpCAM+ cells were CD44+, indicating that CD44 is almost equivalent to EpCAM as a CTC marker and therefore it is not useful to differentiate subpopulations of CTCs in ESCC.

The most widely studied CTC detection method is an immunomagnetic-based enrichment, requiring fixation of the cells, using EpCAM and cytokeratin antibodies for subsequent immunological identification [5, 6]. Previous studies on the detection of CTCs in ESCC have also used a CELLSEARCH system, which allows immunomagnetic capture of fixed EpCAM+ cells in combination with cytokeratin and CD45 immunofluorescence staining [28, 29]. In contrast, we used flow cytometry to detect CTCs, which enabled us to analyze the expression of multiple cell surface markers in viable cells. We sorted an EpCAM + p75NTR+ CTC and cytologically identified an atypical cell as well.

However, we could not obtain primary culture of sorted EpCAM + p75NTR+ CTC from any of the 23 examined patients and thus could not assess the biological properties of the cells. A recent report from our laboratory demonstrated that 100 and 3000 cells were minimally required to establish colonies in vitro and mouse xenograft tumors, respectively, using p75NTR+ cells isolated from cultured KYSEs by flow cytometric cell sorting, suggesting decreased cell survival and recovery due to cell damage during isolation procedure [18].

Another possibility is that a small part (1/3000–1/100) of p75NTR+ cells are true CSCs which can establish colonies or xenograft tumors. In addition, a previous report demonstrated that even using resected primary tumor, only 21 esophageal cancer cell lines (KYSEs) were established from 50 ESCC patients with a success rate of 42 % in the same culture condition which we used in this study [20]. Taken together, improvement of devices, procedure, and culture condition, which can enrich and recover target cells without causing cell damage or cell death, is needed to establish CTC isolation with higher yield and higher viability. Thereafter, characterization of the isolated EpCAM + p75NTR+ CTCs may provide us with a better understanding of their biological features in ESCC.

A proportion of EpCAM+ cells was detectable, even in controls, although the cell counts were significantly lower than in ESCC patients. The presence of these cells could possibly be explained by a nonspecific immunological reaction or contamination of skin cells [30]. In addition, a previous study in mice demonstrated the presence of EpCAM+ bone marrow-derived mesenchymal stem cells with expression of epithelial phenotypic markers [31], suggesting that detection of EpCAM+ non-cancer cells is possible in the peripheral blood. Again, characterization of isolated EpCAM+ cells from the peripheral blood of healthy controls may also provide us with a better understanding of the biological significance of EpCAM+ cells.

The presence of EpCAM + p75NTR+ CTCs did not correlate with p75NTR expression in the primary ESCC tumors, demonstrating the heterogeneity of cell surface marker expression, as has been shown in various other types of tumors [32]. In addition, regulation of p75NTR expression by various environmental stimuli, including tissue injury and pro-inflammatory cytokines, has been reported [33]. This may suggest possible induction of p75NTR expression after mobilization of the metastatic cancer cells into systemic circulation.

## Conclusions

Our flow cytometric detection of CTCs in patients with ESCC has highlighted the clinical significance of EpCAM + p75NTR+ CTCs in ESCC, providing us with a basis from which to establish large-scale prospective studies. Further investigations based on the isolation of viable EpCAM + p75NTR+ CTCs may shed light on the molecular and biological roles of these cells in tumor metastasis in ESCC as well.

### Consent for publication

Not applicable.

## Abbreviations

p75NTR: p75 neurotrophin receptor
CSCs: cancer stem cells
ESCC: esophageal squamous cell carcinoma
CTCs: circulating tumor cells
CTSCs: circulating tumor stem cells
EpCAM: epithelial cell adhesion molecule

## References

[1] Kamangar F, Dores GM, Anderson WF (2006). Patterns of cancer incidence, mortality, and prevalence across five continents: defining priorities to reduce cancer disparities in different geographic regions of the world. J Clin Oncol.

[2] Rice TW, Rusch VW, Apperson-Hansen C, Allen MS, Chen L-Q, Hunter JG (2009). Worldwide esophageal cancer collaboration. Dis Esophagus.

[3] Thallinger CMR, Raderer M, Hejna M (2011). Esophageal cancer: a critical evaluation of systemic second-line therapy. J Clin Oncol.

[4] Hughes AD, King MR (2012). Nanobiotechnology for the capture and manipulation of circulating tumor cells. Wiley Interdiscip Rev Nanomed Nanobiotechnol.

[5] Ross AH, Herlyn D, Iliopoulos D, Koprowski H (1986). Isolation and characterization of a carcinoma-associated antigen. Biochem Biophys Res Commun.

[6] Mostert B, Sleijfer S, Foekens JA, Gratama JW (2009). Circulating tumor cells (CTCs): detection methods and their clinical relevance in breast cancer. Cancer Treat Rev.

[7] Cohen SJ, Punt CJA, Iannotti N, Saidman BH, Sabbath KD, Gabrail NY (2008). Relationship of circulating tumor cells to tumor response, progression-free survival, and overall survival in patients with metastatic colorectal cancer. J Clin Oncol.

[8] Cristofanilli M, Budd GT, Ellis MJ, Stopeck A, Matera J, Miller MC (2004). Circulating tumor cells, disease progression, and survival in metastatic breast cancer. N Engl J Med.

[9] De Bono JS, Scher HI, Montgomery RB, Parker C, Miller MC, Tissing H (2008). Circulating tumor cells predict survival benefit from treatment in metastatic castration-resistant prostate cancer. Clin Cancer Res.

[10] Reya T, Morrison SJ, Clarke MF, Weissman IL (2001). Stem cells, cancer, and cancer stem cells. Nat Publ Group.

[11] Clarke MF, Dick JE, Dirks PB, Eaves CJ, Jamieson CHM, Jones DL (2006). Cancer stem cells—perspectives on current status and future directions: AACR Workshop on cancer stem cells. Cancer Res.

[12] Dean M, Fojo T, Bates S (2005). Tumour stem cells and drug resistance. Nat Rev Cancer.

[13] Schatton T, Frank MH (2008). Cancer stem cells and human malignant melanoma. Pigment Cell Melanoma Res.

[14] Grover PK, Cummins AG, Price TJ, Roberts-Thomson IC, Hardingham JE (2014). Circulating tumour cells: the evolving concept and the inadequacy of their enrichment by EpCAM-based methodology for basic and clinical cancer research. Ann Oncol.

[15] Wicha MS, Hayes DF (2011). Circulating tumor cells: not all detected cells are bad and not all bad cells are detected. J Clin Oncol.

[16] Okumura T, Tsunoda S, Mori Y, Ito T, Kikuchi K, Wang TC (2006). The biological role of the low-affinity p75 neurotrophin receptor in esophageal squamous cell carcinoma. Clin Cancer Res.

[17] Huang S-D, Yuan Y, Liu X-H, Gong D-J, Bai C-G, Wang F (2009). Self-renewal and chemotherapy resistance of p75NTR positive cells in esophageal squamous cell carcinomas. BMC Cancer.

[18] Yamaguchi T, Okumura T, Hirano K, Watanabe T, Nagata T, Shimada Y, Tsukada K. p75 neurotrophin receptor expression is a characteristic of the mitotically quiescent cancer stem cell population present in esophageal squamous cell carcinoma. Int J Oncology. 2016 (in press). 10.3892/ijo.2016.3432 26984177

[19] Zhao JS, Li WJ, Ge D, Zhang PJ, Li JJ, Lu CL (2011). Tumor initiating cells in esophageal squamous cell carcinomas express high levels of CD44. PLoS One.

[20] Shimada Y, Imamura M, Wagata T, Yamaguchi N, Tobe T (1992). Characterization of 21 newly established esophageal cancer cell lines. Cancer.

[21] Iinuma H, Watanabe T, Mimori K, Adachi M, Hayashi N, Tamura J (2011). Clinical significance of circulating tumor cells, including cancer stem-like cells, in peripheral blood for recurrence and prognosis in patients with Dukes’ stage B and C colorectal cancer. J Clin Oncol.

[22] Wang N, Shi L, Li H, Hu Y, Du W, Liu W (2012). Detection of circulating tumor cells and tumor stem cells in patients with breast cancer by using flow cytometry: a valuable tool for diagnosis and prognosis evaluation. Tumour Biol.

[23] Baccelli I, Schneeweiss A, Riethdorf S, Stenzinger A, Schillert A, Vogel V (2013). Identification of a population of blood circulating tumor cells from breast cancer patients that initiates metastasis in a xenograft assay. Nat Biotechnol.

[24] Pilati P, Mocellin S, Bertazza L, Galdi F, Briarava M, Mammano E (2012). Prognostic value of putative circulating cancer stem cells in patients undergoing hepatic resection for colorectal liver metastasis. Ann Surg Oncol.

[25] Sun Y-F, Xu Y, Yang X-R, Guo W, Zhang X, Qiu S-J (2013). Circulating stem cell-like epithelial cell adhesion molecule-positive tumor cells indicate poor prognosis of hepatocellular carcinoma after curative resection. Hepatology.

[26] Theodoropoulos PA, Polioudaki H, Agelaki S, Kallergi G, Saridaki Z, Mavroudis D (2010). Circulating tumor cells with a putative stem cell phenotype in peripheral blood of patients with breast cancer. Cancer Lett.

[27] Li M, Zhang B, Zhang Z, Liu X, Qi X, Zhao J (2014). Stem cell-like circulating tumor cells indicate poor prognosis in gastric cancer. Biomed Res Int.

[28] Reeh M, Effenberger KE, Koenig AM, Riethdorf S, Eichstädt D, Vettorazzi E (2015). Circulating tumor cells as a biomarker for preoperative prognostic staging in patients with esophageal cancer. Ann Surg.

[29] Tanaka M, Takeuchi H, Osaki Y, Hiraiwa K, Nakamura R, Oyama T (2014). Prognostic significance of circulating tumor cells in patients with advanced esophageal cancer. Esophagus.

[30] Paterlini-Brechot P, Benali NL (2007). Circulating tumor cells (CTC) detection: clinical impact and future directions. Cancer Lett.

[31] Okumura T, Wang SS, Takaishi S, Tu SP, Ng V, Ericksen RE (2009). Identification of a bone marrow-derived mesenchymal progenitor cell subset that can contribute to the gastric epithelium. Lab Invest.

[32] Dick JE (2008). Stem cell concepts renew cancer research. Blood.

[33] Choi S, Friedman WJ (2009). Inflammatory cytokines IL-1β and TNF-α regulate p75NTR expression in CNS neurons and astrocytes by distinct cell-type-specific signaling mechanisms. ASN Neuro.

